# Modification and validation of the Endopep-mass spectrometry method for botulinum neurotoxin detection in liver samples with application to samples collected during animal botulism outbreaks

**DOI:** 10.1007/s00216-020-03001-z

**Published:** 2020-10-29

**Authors:** Annica Tevell Åberg, Ida Karlsson, Mikael Hedeland

**Affiliations:** 1 Department of Chemistry, Environment, and Feed Hygiene, National Veterinary Institute (SVA), 751 89 Uppsala, Sweden; 2 Department of Medicinal Chemistry, Analytical Pharmaceutical Chemistry, Faculty of Pharmacy, Uppsala University, P.O. Box 574, 751 23 Uppsala, Sweden

**Keywords:** Endopep-MS, Botulism, Botulinum neurotoxin, BoNT, Liver, Protease inhibitor

## Abstract

Botulinum neurotoxins (BoNTs) are the most potent toxins known and they cause the paralytic disease botulism in humans and animals. In order to diagnose botulism, active BoNT must be detected in biological material. Endopep-MS is a sensitive and selective method for serum samples, based on antibody capture, enzymatic cleavage of target peptides, and detection of cleavage products using matrix-assisted laser desorption ionization-time-of-flight mass spectrometry (MALDI-TOF MS). In many cases of animal botulism, serum samples are not available or they do not contain detectable amounts of BoNT and liver sampling is an alternative for postmortem examinations. However, the Endopep-MS method is impaired by the inherent protease activity of liver samples. In the presented study, the Endopep-MS method has been successfully modified and validated for analysis of cattle, horse, and avian liver samples, introducing a combination of a salt washing step and a protease inhibitor cocktail. These modifications resulted in a substantial decrease in interfering signals and increase in BoNT-specific signals. This led to a substantial improvement in sensitivity for especially BoNT-C and C/D which are among the most prominent serotypes for animal botulism. Botulism was diagnosed with the new method in liver samples from dead cattle and birds from outbreaks in Sweden.

**Electronic supplementary material:**

The online version of this article (10.1007/s00216-020-03001-z) contains supplementary material, which is available to authorized users.

**Figure:**
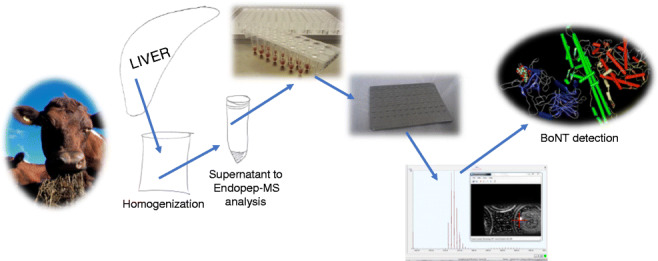
Graphical Abstract

## Introduction

The paralytic disease botulism is caused by botulinum neurotoxins (BoNTs) which are produced by anaerobic bacteria, mainly *Clostridium botulinum*. There are several known serotypes of BoNTs which are denoted A, B, C, D, E, F, G, and X [1–3]. All of the BoNTs are around 150 kDa large proteins that consist of a heavy chain, responsible for the transport across the neuronal membrane into the nerve cell, and a light chain, responsible for the toxic zinc metalloprotease activity. Inside the nerve cell, the light chain of BoNT cleaves one of the three proteins essential for the soluble *N*-ethylmaleimide-sensitive factor activating protein receptor (SNARE) complex formation, resulting in inhibition of the release of acetyl choline into the synaptic cleft leading to flaccid paralysis [4–6].

Botulism affects both humans and animals. Human botulism is normally associated with BoNT-A, B, E, and F [6], while horses are susceptible to BoNT-A, B, or C [7]. Cattle botulism outbreaks are caused by BoNT-B, C, D, or the mosaic forms thereof called C/D or D/C [8], and botulism in minks [9], and wild and domestic birds usually derive from BoNT-C, D, C/D, or D/C [10]. The disease could either develop after the consumption of food or feed containing the preformed toxin, or by so-called toxicoinfection when the bacteria grow and produce the toxin inside the body, e.g., in the intestine (birds, foals, infants) or in a wound (syringe using drug addicts) [6, 7].

BoNTs are the most toxic compounds known; hence, a very small amount is needed to cause disease. But it also means that methods used to detect BoNTs need to be very sensitive, in order to measure such low concentrations present in a biological sample (i.e., in the order of pg/mL). The traditionally used method is the mouse bioassay (MBA) [11], but because of both practical and ethical issues, it needs to be replaced [12]. Alternative methods that do not require laboratory animals are for example polymerase chain reaction (PCR), enzyme-linked immunosorbent assay (ELISA), and the endopeptidase mass spectrometric method called Endopep-MS. Prior to PCR analysis, the bacteria in the sample need to be anaerobically cultivated, and then the toxin gene can be detected [13–16]. It is a fast and cheap method, and it works well for detection as long as spores and/or vegetative cells of the bacteria are present in the sample, i.e., the test will be negative if only preformed BoNT is present. Furthermore, the detection of the toxin gene does not prove that there are viable cells or expressed toxin in the sample. ELISAs for BoNT detection can be designed in a variety of ways [17–20]. Regular ELISA is very sensitive and specific but will not differentiate between active and inactive toxin, e.g., BoNT which has been denatured or where the heavy and light chains have been separated. Endopeptidase immunoassay is activity-based [21, 22], and will only detect active BoNT, but when it comes to biological samples, matrix components can affect the detection and might result in false positive or negative results. The Endopep-MS method is also activity-based, and detects only the active toxin [23, 24]. The BoNT in a sample is captured by antibodies selective towards the heavy chain of the BoNT [24]. The antibodies are attached to magnetic beads and, by the use of a magnet, the beads and captured BoNTs can be removed from the matrix, washed, and then released into a buffer solution containing synthetic peptides that mimic the amino acid sequence of one of the SNARE proteins. If BoNT is present in the sample, the peptide will be cleaved by the BoNT light chain in a specific position and the mass-to-charge ratio (*m/z*) of the cleavage products can be measured by matrix-assisted laser desorption ionization time of flight (MALDI-TOF) mass spectrometry. The Endopep-MS method has been validated for several different types of samples, e.g., human and chicken serum, milk, food, and human stool [24–27].

In cases of cattle and horse botulism, serum is almost always negative when the botulism symptoms manifest [28, 29], probably because the BoNT by then has left the blood stream and entered the nerve cells. In order to detect BoNT in these cases, other types of samples might be needed. Liver is often collected postmortem and PCR analysis has demonstrated the presence of the toxin gene after anaerobic cultivation in both cattle and avian botulism cases [29, 30]. The mouse bioassay has also detected BoNT in liver samples from cattle [28], meaning that both the toxin and spores of the Clostridia are sometimes present. In liver samples that contain endogenous proteases, there might be an interference with the Endopep-MS BoNT detection in two different ways: cleavage of the peptide used for detection in the same position as the BoNT, leading to false positive results, or cleavage of the peptide in other positions resulting in false negative results. Thus, there is a great need to improve the Endopep-MS method to also encompass liver samples. Previously, problems with unspecific protease activity in human stool samples have been overcome using a salt washing step and the protease inhibitor antipain for detection of BoNT/A [31].

The aim of this study was to modify and validate the Endopep-MS method for detection of BoNT-C, C/D, D, and D/C in liver samples from birds, horses, and cattle. The problem of inherent sample protease activity was circumvented by using a salt washing step in combination with a mixture of protease inhibitors. Finally, the new method was used to analyze naturally contaminated cattle and avian liver samples collected during different botulism outbreaks.

## Materials and methods

All experiments with botulinum neurotoxins were carried out in a class 2 biosafety cabinet with HEPA filters.

### Chemicals

HEPES-buffered saline solution with EDTA and surfactant P20 pH 7.4 (HBS-EP buffer) were obtained from GE Healthcare (Uppsala, Sweden); phosphate-buffered saline (PBS), zinc chloride (ZnCl_2_), α-cyano-4-hydroxy cinnamic acid (CHCA), dithiothreitol (DTT), ammonium citrate, bovine serum albumin (BSA), Tween^®^20, trifluoroacetic acid (TFA), and protease inhibitor cocktail P8340 (consisting of 104 mM AEBSF or 4-(2-aminoethyl)benzenesulfonyl fluoride, 80 μM aprotinin, 4 mM bestatin, 1.4 mM E-64, 2 mM leupeptin, and 1.5 mM pepstatin A) were all purchased from Sigma-Aldrich (St. Louis, MO, USA). Dynabeads M-280 Streptavidin, and sulfo-NHS-biotin, No-Weight™ were purchased from Life Technologies (Stockholm, Sweden) and Thermo Scientific (Waltham, MA, USA), respectively. Monoclonal antibodies used for BoNT extraction were RAZ1 and B12.2 (selective towards A and B heavy chain, respectively), 6F5.1 (selective towards both F and E heavy chain) 8DC1.2 (selective towards both C and D heavy chain), and 1C1.1 (selective towards C heavy chain only) were purchased from Dr. James Marks at the University of California (San Francisco, USA). Botulinum neurotoxins A, B, C, D/C, E, and F complexes were obtained from Metabiologics (Madison, WI, USA) and used at approximately 2.5 MLD_50_ for BoNT-A, B, E, and F, i.e., 0.071 ng (BoNT-A), 0.28 ng (BoNT-B), 17 ng (BoNT-E), and 0.50 ng (BoNT-F), and approximately 10 MLD_50_ for BoNT-C, and D/C, i.e., 1.7 ng (BoNT-C) and 0.33 ng (BoNT-D/C) per 500 μL sample. Purified BoNT-C/D and D produced recombinantly in *Escherichia coli* was supplied from Toxogen GmbH (Hanover, Germany) and used at approximately 1.6 and 0.3 ng per 500 μL sample, respectively. The substrate peptides used for BoNT-A, B, C, D, E, F, and as internal standard (see Table 1) were purchased from Xaia Custom peptides (Gothenburg, Sweden) at a purity of ≥ 98%. The water used was purified with a Milli-Q water purification system (Millipore, Bedford, MA, USA) and all other chemicals were of analytical grade or better and used without further purification.

**Table 1:** Amino acid sequences and cleavage sites for the peptide substrates used in the Endopep-MS reaction

BoNT serotype	Substrate peptide and cleavage products	*m/z*
A	Ac-RGSNKPKIDAGN**QR**ATRXLGGR-NH_2_	2406.4
	Ac-RGSNKPKIDAGNQ	1426.7
	RATRXLGGR-NH_2_	998.6
B	LSELDDRADALQAGAS**QF**ESSAAKLKRKYWWKNLK	4024.1
	LSELDDRADALQAGASQ	1759.9
	FESSAAKLKRKYWWKNLK	2283.3
C and C/D	Ac-VKYNIDEAQN**KA**S-Ornithine-MGIRRR-NH_2_	2405.3
	Ac-VKYNIDEAQNK	1363.7
	AS-Ornithine-MGIRRR-NH_2_	1059.6
D and D/C	H_2_N-LQQTQAQVDEVVDIMRVNVDKVLERDQ**KL**SELDDRADAL-OH	4498.0
	H_2_N-LQQTQAQVDEVVDIMRVNVDKVLERDQK	3297.7
	LSELDDRADAL-OH	1217.6
E	WWWAKLGQEIDTRNRQKD**hRI**MAKADSNKR-NH_2_	3611.9
	WWWAKLGQEIDTRNRQKDhR	2498.3
	IMAKADSNKR-NH_2_	1132.6
F	TSNRRLQQTQAQVDEVVDIMRVNVDKVLERD**QK**LSELDDRADALQAGAS	5523.8
	TSNRRLQQTQAQVDEVVDIMRVNVDKVLERDQ	3783.0
	KLSELDDRADALQAGAS	1759.9
IS (internal standard)	LRTAQADITNSK-Biotin	1543.8

Bold characters indicate cleavage site for the toxin. *Ac* acetylated terminus, *NH*_*2*_ amidated terminus, *X* norleucine, *hR* homo arginine

The Endopep-MS reaction buffer without protease inhibitor cocktail consisted of 18 μL HEPES buffer (20 mM, pH 7.3) with 200 μM ZnCl_2_ and 1.0 mg/mL BSA, and 1.0 μL DTT (10 mM) and 1.0 μL substrate peptide (50 μM) for each sample. When protease inhibitor cocktail was used, the P8340 solution was first diluted ten times in HEPES buffer (20 mM, pH 7.3) with 200 μM ZnCl_2_ and 1.0 mg/mL BSA and then 2–8 μL per sample was added to the 20 μL of Endopep-MS reaction buffer.

The CHCA MALDI-matrix was dissolved in Milli-Q water/acetonitrile/1 M ammonium citrate (aq)/10% TFA (aq) (98/98/2/2, v/v/v/v) at a concentration of 5.0 mg/mL. A total of 18 μL of the matrix was mixed with 2.0 μL of the internal standard peptide (0.1 nmol) for each sample.

### Sample collection

The liver samples used in the method development and validation studies were obtained as clinical samples PCR negative for BoNTs (turkey) or purchased from local slaughter houses (cattle and horse). The suspected botulism cases analyzed by the new Endopep-MS protocol were all delivered to the Swedish National Veterinary Institute (SVA) for postmortem examination. During the necropsy, samples were recovered for different routine examinations and the liver samples were sent to Endopep-MS analysis.

### Sample preparation

The liver samples, both clinical samples and the samples for development and validation of the method, were rinsed with PBST buffer and then placed in Stomacher^®^80 standard filter bags. One milliliter of PBST buffer was added per gram of liver and the samples were homogenized by a Stomacher^®^80 Biomaster from Seward, Worthing, UK [27]. The liquid from the stomacher bags was poured into 50 mL centrifugal tubes and were centrifuged for 20 min at 4 °C and 3756*g*. The supernatant was stored at − 20 °C until analysis. After defrosting, the samples were centrifuged again for 10 min at room temperature and 3756*g* in order to obtain a clear supernatant. Five hundred microliter of the supernatant was used for Endopep-MS analysis. To prepare the spiked samples, the active BoNT was added into the 500 μL of liver homogenate supernatant in the 96 deep well plate.

### The regular Endopep-MS protocol

The antibodies (0.5 mg/mL in HBS-EP buffer) were biotinylated over night with 1.0 μL of 1.0 mM sulfo-NHS-biotin solution per 5 μg antibody. The next day, 5 μg of antibodies were incubated for 1 h at room temperature in 250 μL of Dynabeads M-280 Streptavidin in Protein LoBind Eppendorf tubes. To a 500 μL liver sample, 20 μL of Dynabeads with coupled antibodies were used to extract botulinum neurotoxin. The extraction of BoNTs from the sample matrix was automatically carried out in 96 deep well plates on a KingFisher Flex (Thermo Scientific, Waltham, MA, USA). After 1-h incubation, the beads were washed in 1 mL of HBS-EP buffer twice, and 150 μL of water once, before resuspension in 150 μL of water. The solution was transferred to a 0.2-mL well in a 24-well PCR plate (Thermo Fisher Scientific, Gothenburg, Sweden) placed in a DynaMag™-96 Side magnetic stand (Life Technologies AS, Oslo, Norway). The water was removed and the Endopep reaction buffer was added. The plates were vortexed and incubated at 42 °C (for BoNT-C and D) or 37 °C (for BoNT-A, B, E, and F) in an Arktik PCR thermo cycler (Thermo Fisher Scientific, Gothenburg, Sweden).

After incubation, for 3 or 21 h, the PCR tubes were placed in the magnetic stand and a 2 μL aliquot was transferred to a new PCR tube containing 18 μL of MALDI-matrix with IS. After vortex mixing, 1.0 μL was spotted onto a stainless steel MALDI target plate. Each sample was spotted in triplicate. The samples were analyzed on a Synapt G2 MALDI-Q-TOF mass spectrometer from Waters Corporation, MA, USA. The MALDI parameters were the same as described in Björnstad et al. [25]. In short, the instrument was operated in MS resolution mode at a positive potential in a mass range of 100–8000 *m/z*. The laser firing rate was 1000 Hz, the scan time was 1.0 s, and the acquisition time was 30 s per spot. Each spectrum was the result of 1000 laser shots and was processed with background subtraction and the automatic peak detection option in the MassLynx V4.1 software (Waters Corporation, MA, USA). The instrument was calibrated by the use of spots of red phosphorus in the range of *m/z* 100–800.

### Modification of the Endopep-MS protocol for liver samples

To evaluate the effect of different approaches on the unspecific cleavage of the peptide substrates used for detection of BoNT-C, C/D, D, and D/C, the C substrate, which was most susceptible to proteases, was selected. Different homogenized samples of avian liver were analyzed by Endopep-MS and the ones that resulted in the most unspecific cleavage of the C substrate were selected for the modification study. The regular Endopep-MS protocol is described above. The salt wash protocol included a modification after 60 min of mixing the beads in the sample; the magnetic beads were washed 2 × 15 s in 2.0 M of sodium chloride in PBST, followed by two washing steps in regular PBST. The protease inhibitor cocktail used was first diluted as described in the *Chemicals* section. Then, different volumes of the diluted solution were added to the prepared Endopep reaction buffer; the total volume was not adjusted and hence differed between experiments. Blank samples and samples spiked with BoNT-C and C/D were analyzed by the regular protocol, with the additional salt wash, with the addition of different volumes of protease inhibitor cocktail in the Endopep reaction buffer, and with the combination of salt wash and addition of protease inhibitors. The results were compared both from the aspect of most efficient inhibition of unwanted protease activity, and from the aspect of not inhibiting the BoNT activity. The inhibition of unspecific protease cleavage was evaluated by measurement of the intensity of the two major unwanted cleavage products of the C-substrate at *m/z* 2250 and *m/z* 2094, and the BoNT activity was evaluated by measurement of the expected cleavage products for BoNT-C, i.e., *m/z* 1059 and *m/z* 1363.

### Validation of the modified Endopep-MS protocol

After method modification, the new improved Endopep-MS protocol was validated. The samples were analyzed as blank and spiked with BoNT-C, C/D, D, or D/C, as six replicates each for turkey liver and four replicates each for cattle, and horse liver. All samples were analyzed after 3 and 21 h of incubation. The inhibition of unspecific protease cleavage was evaluated by comparing the normalized intensity of the peaks for the two unwanted cleavage products of the C-substrate at *m/z* 2250 and *m/z* 2094 in the spectra from the regular and the modified protocols. The BoNT activity was evaluated by measuring the normalized intensity of the peaks of the expected cleavage products, i.e., *m/z* 1059 and *m/z* 1363 for BoNT-C, and C/D, or *m/z* 1218 and *m/z* 3298 for BoNT-D and D/C, in the spiked samples compared to the blank control samples. The spectra from the regular and the modified protocols were evaluated. More than 3× the blank signal was considered a positive sample [25].

For cattle liver, the BoNT-A, B, E, and F activities were also evaluated by measuring the expected cleavage products: *m/z* 998 and 1426 for BoNT-A, *m/z* 1759 and *m/z* 2283 for BoNT-B, *m/z* 1132 and *m/z* 2500 for BoNT-E, and *m/z* 1759 and *m/z* 3783 for BoNT-F. Three replicates of blank and spiked samples (2.5 and 5 MLD_50_ of BoNT-A, B, E, and F, respectively) were analyzed by the two different protocols.

## Results and discussion

**Fig. 1:**
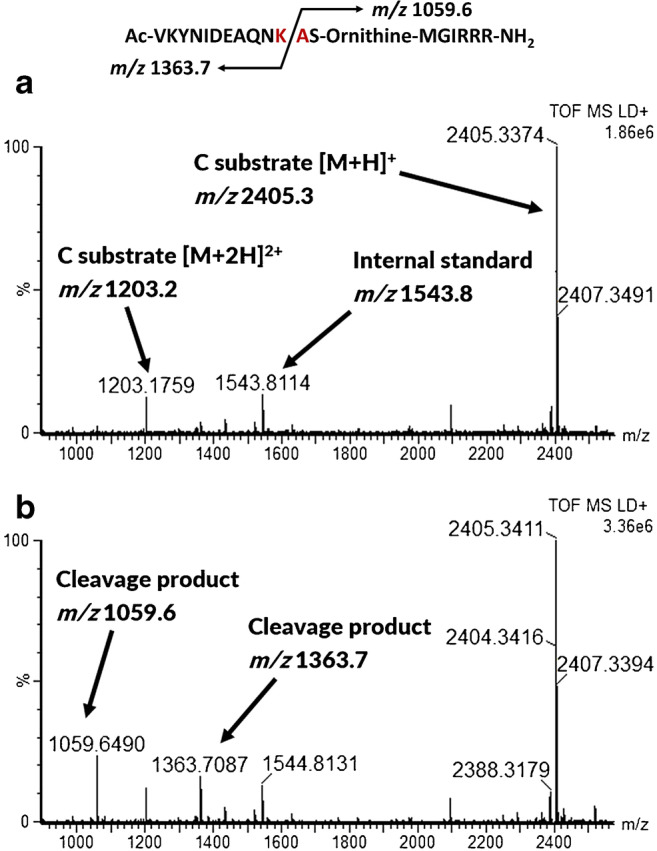
Illustration of the BoNT-C cleavage site of the C peptide, and spectra of blank chicken serum (**a**) and chicken serum spiked with 5 MLD_50_ BoNT-C (**b**), both analyzed by the regular Endopep-MS protocol

**Fig. 2:**
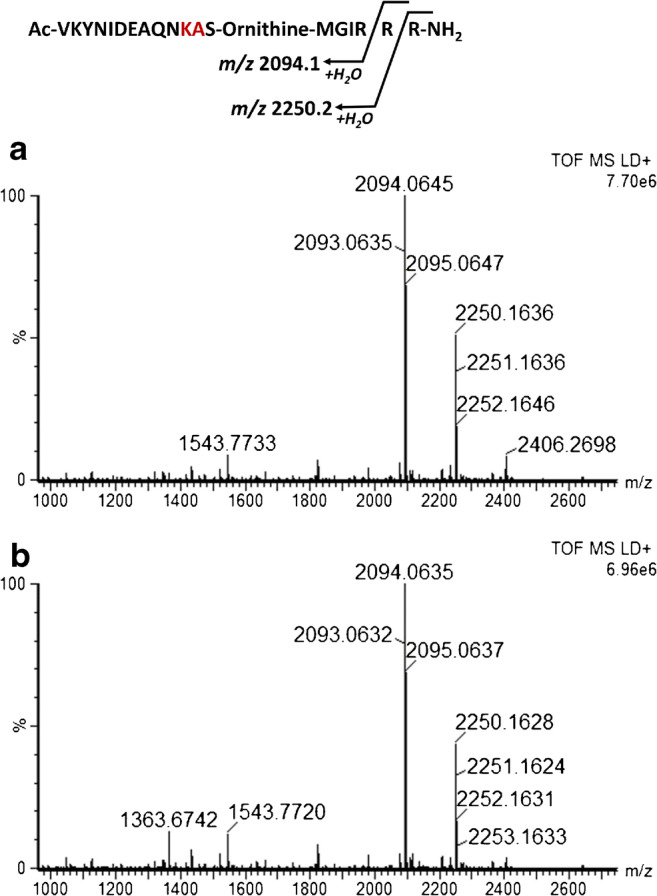
Mass spectra of blank turkey liver (**a**) and turkey liver spiked with 10 MLD_50_ BoNT-C (**b**) analyzed with the regular Endopep-MS protocol. The C peptide substrate is almost completely cleaved into *m/z* 2094.1 and 2250.2 in both the blank and the spiked sample, and in the spiked sample, only one of the expected BoNT-C cleavage products, *m/z* 1363.7, is visible. The peak at *m/z* 1543.8 represents the internal standard

The activity-based Endopep-MS method, which is very sensitive and selective in serum, is severely impaired when used with liver samples, owing to their inherent protease activity. Figure 1a illustrates the MALDI-TOF mass spectrum of a blank chicken serum sample analyzed with the original Endopep-MS method [25], where the peptide substrate remains uncleaved. The peak at *m/z* 1543.8 represents the internal standard and the peaks at *m/z* 2405.3 and *m/z* 1203.2 represent the intact singly and doubly charged BoNT-C substrate, respectively. Figure 1b represents a mass spectrum of a blank chicken serum sample spiked with BoNT-C, and the protease activity of BoNT-C results in the two expected cleavage products at *m/z* 1059.6 and *m/z* 1363.7. Figure 2a and b illustrate a turkey liver homogenate sample after 21 h of incubation analyzed blank and spiked with BoNT-C, respectively. After incubation, the C peptide substrate has been cleaved into mainly two unspecific cleavage products: *m/z* 2094.1 and 2250.2, respectively, and the peptide substrate is almost completely consumed because of the presence of endogenous proteases. The major intended cleavage product at *m/z* 1059.6 is not visible at all (see Fig. 2b) and the presence of the unspecific products make the spectrum difficult to interpret and it is impossible to use Endopep-MS to detect the presence of BoNTs in the liver sample.

### Method development

One possible approach to prevent the unspecific cleavage of the peptide substrates is a more extensive washing procedure to remove proteases other than BoNTs from the magnetic beads. Extra washing steps with 2.0 M sodium chloride prior to the endopeptidase reaction has previously been shown effective for BoNT-A detection in human stool samples [31]. Another approach is to inhibit the unwanted proteases in the Endopep reaction step, but without inhibiting the BoNT activity, which has been previously evaluated for BoNT-A [31], B, E, and F in stool [24]. However, the addition of protease inhibitor cocktails resulted in undesired inhibition of the BoNT activity but the single compound antipain proved useful.

In the present study, we aimed for the first time to develop the Endopep-MS method for detection of BoNTs in liver samples which have a high protease content. The unspecific proteolysis has been demonstrated to be most problematic for determination of BoNT-C and the mosaic C/D, which are among the most important serotypes for animal botulism. A combination of protease inhibitors was evaluated. The cocktail used, P8340, consists of a combination of substances that each has specific inhibitory properties. AEBSF and aprotinin act to inhibit serine proteases, including trypsin, chymotrypsin, and plasmin among others. Bestatin inhibits aminopeptidases, E-64 acts against cysteine proteases, leupeptin acts against both serine and cysteine proteases, and finally pepstatin A inhibits acid proteases.

The two different approaches, i.e., salt wash and protease inhibitor addition, separate and in combination, were evaluated for BoNT-C in liver samples. Avian liver samples were selected for their high endogenous protease content, and the C substrate was selected for the initial evaluation, since it is more susceptible to unwanted protease cleavage than the D substrate.

**Fig. 3:**
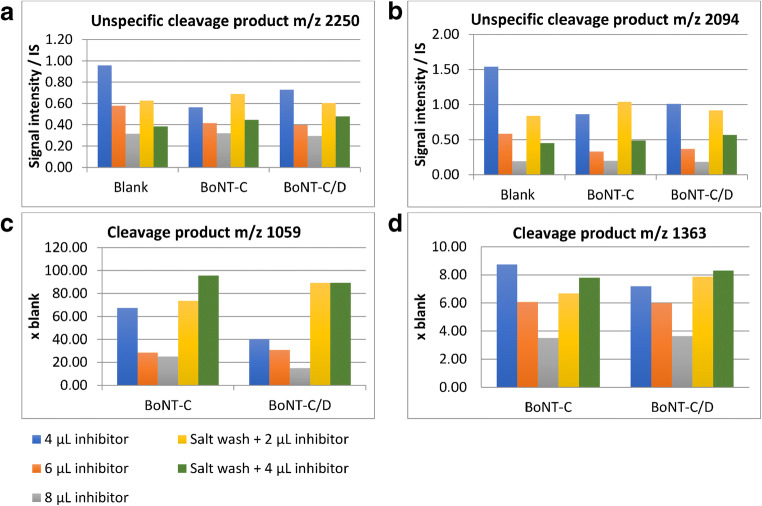
Results from method development, using the Endopep-MS protocol with addition of different volumes of protease inhibitor cocktail alone, or in combination with the salt washing step. Turkey liver homogenate samples were analyzed blank and spiked with BoNT-C or C/D and incubated for 21 h. The intensity of the unwanted cleavage products of the C substrate *m/z* 2250 and 2094 (illustrated in **a** and **b**, respectively), and the two expected peptide cleavage products for BoNT-C and C/D (illustrated in **c** and **d**)

**Fig. 4:**
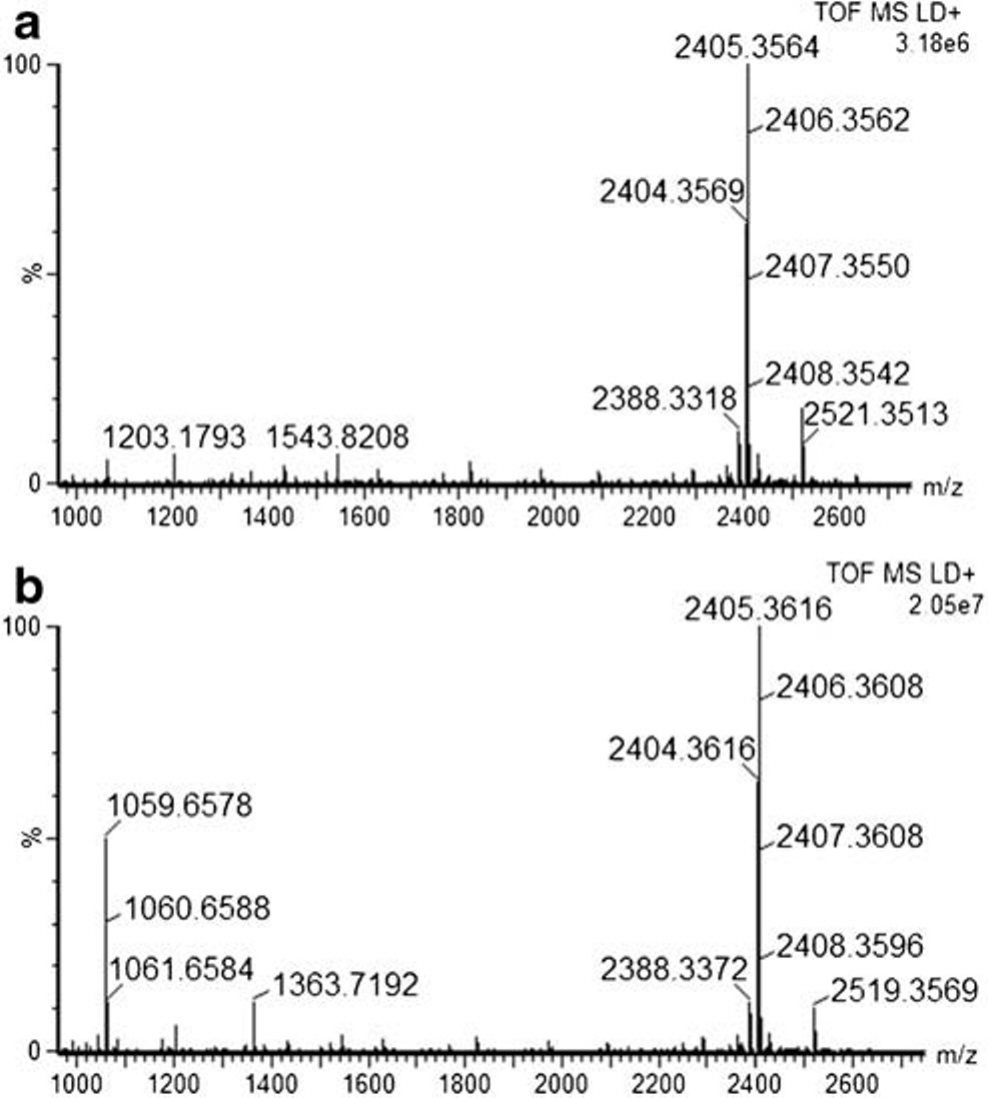
Results from method development of Endopep-MS for turkey liver. The samples were analyzed blank (**a**) and spiked with 10 MLD_50_ of BoNT-C (**b**) by the modified Endopep-MS protocol. The peak at *m/z* 1543.8 represents the internal standard

Salt wash alone did not solve the issues with the endogenous proteases in avian liver (results not shown) but in combination with the addition of protease inhibitor cocktail in the Endopep reaction buffer, the proteases could be effectively inhibited. In Fig. 3a and b, the intensities of the two most dominating unwanted cleavage products, *m/z* 2250 and *m/z* 2094, were compared for three different concentrations of the protease inhibitor cocktail alone, and in combination with the salt wash. The more protease inhibitor added, the more the intensity of both unwanted cleavage products were reduced, and in combination with salt wash, it appeared that less inhibitor could be added in the buffer to achieve comparable protease inhibition. The intensities of the expected cleavage products for BoNT-C, i.e., *m/z* 1059 and *m/z* 1363, respectively, were compared in the same experiment (Fig. 3c and d). The addition of 4 μL or more of the protease inhibitor cocktail to the reaction buffer resulted in an inhibition of the BoNT activity (for concentrations of the individual compounds, see the Experimental section). However, in combination with the salt wash, there was no inhibition of the BoNT-C in turkey liver (c.f. Fig. 3c and d). It was concluded that the combination of salt wash and 4 μL of the protease inhibitor cocktail successfully inhibited unwanted activity from endogenous proteases present in the liver samples without inhibiting the BoNT. Figure 4a and b illustrate the turkey liver homogenate analyzed with the new modified Endopep-MS protocol both blank and spiked with BoNT-C. The mass spectra resulting from the new protocol look similar to those of the chicken serum (c.f. Fig. 1), and much improved compared to the spectra in Fig. 2. Furthermore, this modified Endopep-MS protocol was validated.

### Method validation

**Fig. 5:**
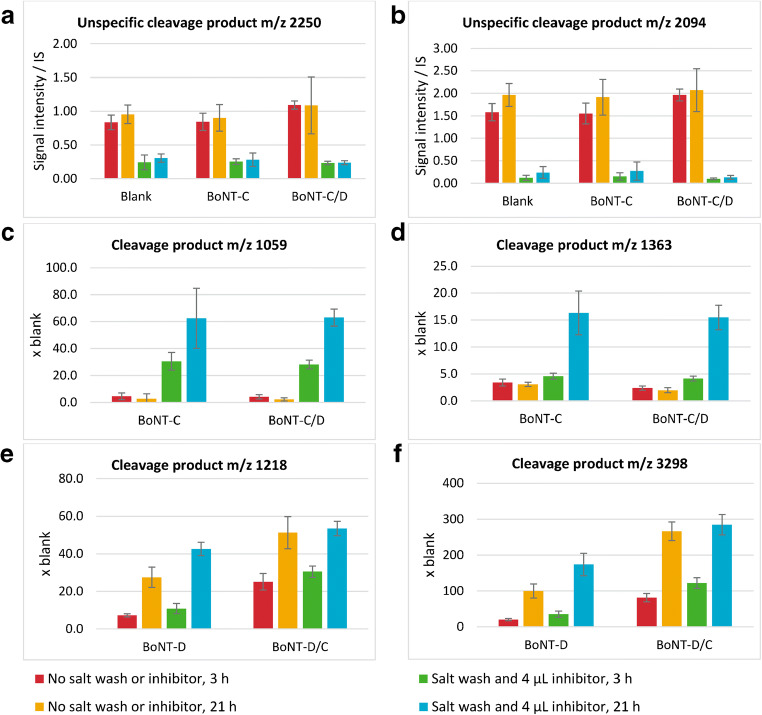
Results from method validation, using the regular and the modified Endopep-MS protocols, at both 3 and 21 h of incubation. Turkey liver homogenate samples were analyzed blank and spiked with BoNT-C, C/D, D, or D/C (six replicates each). The intensity of the unwanted cleavage products of the C substrate *m/z* 2250 and 2094 (illustrated in **a** and **b**, respectively), and the two expected peptide cleavage products for BoNT-C and C/D (illustrated in **c** and **d**) and for BoNT-D and D/C (illustrated in **e** and **f**)

The modified protocol, i.e., with the salt wash in combination with the addition of 4 μL of diluted P8340 protease inhibitor cocktail to the Endopep reaction buffer, was validated for turkey, cattle, and horse liver homogenates. Blank samples and samples spiked with approximately 10 MLD_50_ of BoNT-C, C/D, D, or D/C were analyzed in six replicates each for turkey, and four replicates each for cattle and horse liver. They were extracted both by the regular and the modified protocol, and all samples were analyzed by MALDI-TOF MS after both 3 and 21 h of incubation. The inhibition of unspecific protease cleavage was evaluated by comparison of the intensity of the two major unwanted cleavage products of the C-substrate at *m/z* 2250 and *m/z* 2094, in the samples extracted by the regular and the modified protocols. The new protocol resulted in a major improvement, as illustrated in Fig. 5a and b, where the intensities of the unwanted cleavage products were significantly reduced after both 3 and 21 h of incubation. There was also substantially higher intensities of the peaks for the intact substrate for the modified compared to the original protocol (results not shown).

The BoNT activity was evaluated by measuring the expected cleavage products for BoNT-C and D, i.e., *m/z* 1059, *m/z* 1363, and *m/z* 1218, *m/z* 3298, respectively. As illustrated in Fig. 5c–f, the new protocol did not inhibit the activity of the BoNTs. On the contrary, the inhibition of the competing proteases in the matrix resulted in higher signals from the BoNT activity, and as expected, the BoNT cleavage product intensity increased by increasing incubation time.

Validation samples of cattle and horse liver were also analyzed for BoNT-C, C/D, D, and D/C with the original and modified Endopep-MS protocols. The detrimental effects of endogenous proteases using the original method and the successful results of the salt wash and protease inhibitor cocktail were also obvious for these matrices (see Electronic Supplementary Material (ESM), Figs. S1 and S2). Furthermore, the modified protocol was evaluated for BoNT-A, B, E, and F in cattle liver in order to test if these toxin serotypes were affected by the addition of protease inhibitor. The activity of BoNT-E was not significantly affected using the modified Endopep-MS protocol while the activities of BoNT-A and F were slightly decreased. For BoNT-B, the activity decreased substantially; however, the samples spiked with 2.5 MLD_50_ of BoNT-A, B, E, and F were still all positive after 3-h incubation (see ESM Fig. S3).

In summary, the validation study has shown that the modified Endopep-MS protocol with salt wash and the protease inhibitor cocktail improved the detection sensitivity substantially for BoNT-C and C/D in bird, cattle, and horse liver samples and the sensitivity for BoNT-D and D/C was improved or unchanged. The modified protocol can also be used without a great loss of performance for detection of BoNT-A, E, and F. However, the target peptide for BoNT-C is the more sensitive to unspecific cleavage by endogenous proteases than the other target peptides. Since the signal for BoNT-B was substantially lowered with the new protocol, it is recommended that clinical liver samples are routinely analyzed with both protocols in order to obtain optimal sensitivity for all BoNT serotypes.

### Analysis of samples from avian and cattle botulism outbreaks

In two different outbreaks of suspected avian botulism, dead birds were sent in for necropsy to the National Veterinary Institute (SVA) in Sweden. The first case involved three mallards, of which two were male and one was female (see Table 2, no. 1). In the second case, there were five birds, two of which were mallards, both male, and three were tufted ducks, two female and one male (no. 2). The birds were not grown thin but had water in their lungs indicating that they were weakened, and botulism was suspected. The livers from the three and five birds, respectively, were pooled, homogenized, and analyzed with the improved Endopep-MS protocol. Both samples were found positive for BoNT-C/D since cleavage of the C substrate was observed when the 8DC1.2 antibody (selective towards C and D heavy chain) was used but not when the 1C1.1 antibody (selective towards C heavy chain only). These results are consistent with those of other studies of botulism cases in poultry and wild birds in Sweden where the mosaic BoNT C/D has been demonstrated to be the causative agent [25, 32, 33]; similar results have been found for other European countries [15] and Japan [34].

**Table 2:** Information on clinical samples analyzed, and results from Endopep-MS analysis using the new protocol

	Sampling place	Year	Type	Matrix	Endopep-MS result
1.	Pond	2018	Mallard (*n* = 3)	Liver	BoNT-C/D
2.	Pond	2018	Mallard (*n* = 2) + Tufted duck (n = 3)	Liver	BoNT-C/D
3.	Farm A	2015	Cattle	Liver	BoNT-C
4.	Farm B	2016	Cattle	Serum	Negative
5.	Farm B	2016	Cattle	Liver	BoNT-C
6.	Farm B	2016	Cattle	Liver	BoNT-C

**Fig. 6:**
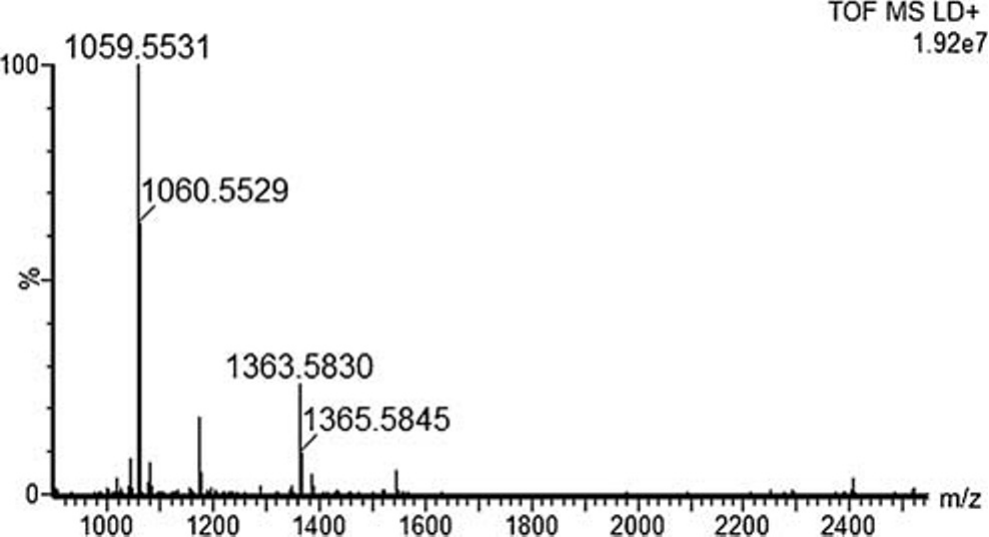
Spectrum of a clinical cattle liver sample (no. 3 in Table 2) analyzed by the modified Endopep-MS protocol. The C peptide substrate is almost completely consumed after 3-h incubation, cleaved into the two cleavage products significant for BoNT-C, i.e., *m/z* 1059.6 and 1363.6. The peak at *m/z* 1543.8 represents the internal standard

Bovine botulism was suspected at two different farms, on two different occasions. The first case involved several animals that had died and poisoning of some kind was suspected. The cattle on the farm fed on both silage and straw. One of the cows (no. 3), female, Swedish red and white, 20 months old, was sent to necropsy and the liver was collected, and homogenized, for Endopep-MS analysis. In the second case, the farmer had found and removed a cadaver in the silage, and shortly thereafter four animals died and two more were found lying down with clinical signs of botulism, i.e., weakness, decreased muscular tonus, and drooling. Serum was collected from one of the sick animals (no. 4) and two of the dead cows were sent to necropsy and liver was collected (nos. 5 and 6). The serum sample was analyzed by the method described by Björnstad et al. [25], but no botulinum neurotoxin could be detected. The two liver samples were homogenized and analyzed with the improved Endopep-MS protocol. High concentration of BoNT-C was detected in all three liver samples, i.e., cleavage of the C substrate was observed both when the 8DC1.2 and the 1C1.1 antibody was used. An example of a mass spectrum is shown in Fig. 6. There are only a few cases of confirmed bovine botulism previously described in Sweden. BoNT-C activity has previously been determined by Endopep-MS in a liver sample from a Swedish black and white cow [35], but it was not concluded whether it was BoNT-C or C/D. BoNT-C has previously been described to cause botulism in cattle in France [36] and Italy [37], although the mosaic BoNT-D/C has been demonstrated to be the most common cause in this species in Italy [38] and France [15].

The results of these authentic case samples demonstrate the usefulness of the improved Endopep-MS protocol to diagnose botulism in liver samples of both diseased birds and cows. The negative serum sample (no. 4) of the cow from the same farm as two of the cows with positive liver samples (nos. 5 and 6) also emphasizes the usefulness of liver analysis. The BoNT concentrations in serum may be below the detection limit when the animal has botulism symptoms [28, 29], because the toxin has left the blood stream and entered the nerve cells at that point.

## Conclusion

The Endopep-MS method was successfully modified for analysis of BoNTs in animal liver samples. The unspecific protease activity inherently present in these samples could be mitigated by a salt wash in combination with addition of a protease inhibitor cocktail. The modified Endopep-MS protocol demonstrated significantly lower degree of unspecific cleavage of target peptides and substantially increased detection sensitivity, especially for BoNT-C and C/D in avian, cattle, and horse liver samples but also for BoNT-D and D/C. Liver sampling and the modified analytical method proved to be very useful to diagnose botulism in dead birds and cows from outbreaks.

## Electronic supplementary material

ESM 1 (PDF 545 kb)
