# Supplementary material for: Modification and validation of the Endopep-mass spectrometry method for botulinum neurotoxin detection in liver samples with application to samples collected during animal botulism outbreaks
Source: Anal Bioanal Chem. 2020 Oct 29;413(2):345–54. doi: 10.1007/s00216-020-03001-z (PMC7806574; doi:10.1007/s00216-020-03001-z)
Supplement: Supplementary file 1 — (PDF 545 kb) [file 216_2020_3001_MOESM1_ESM.pdf]

Analytical and Bioanalytical Chemistry

Electronic Supplementary Material

Modification and validation of the Endopep-mass spectrometry method for botulinum neurotoxin detection in liver samples with application to samples collected during animal botulism outbreaks

Annica Tevell Åberg, Ida Karlsson, Mikael Hedeland

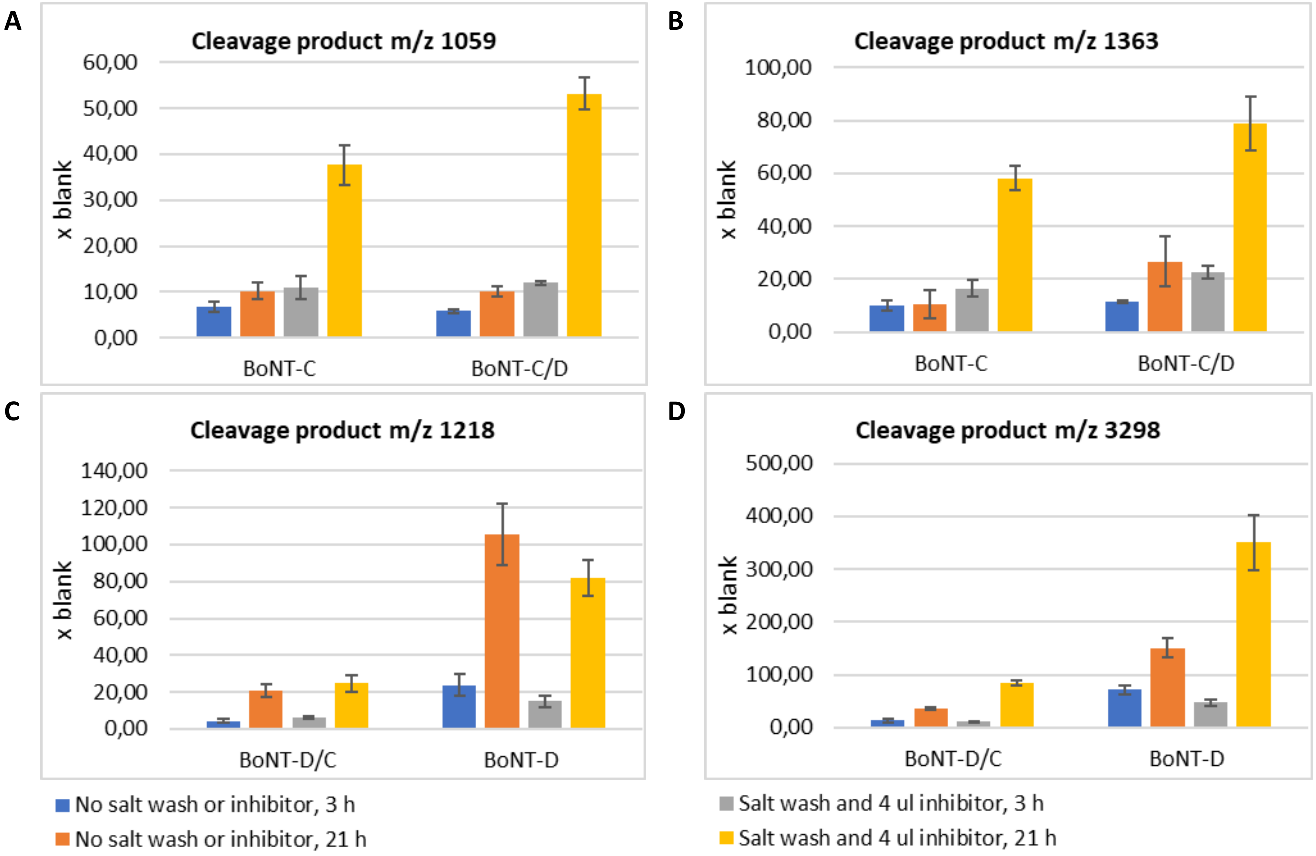

Fig. S1 Results from the method validation, using the regular and the modified Endopep-MS protocols at both 3 and 21 hours of incubation. Cattle liver homogenate samples were analyzed blank and spiked with BoNT-C, C/D, D, or D/C (four replicaties each). The results for the two expected peptide cleavage products for BoNT-C and C/D are illustrated in A and B, respectively, and for BoNT-D/C and D illustated in C and D, respectively

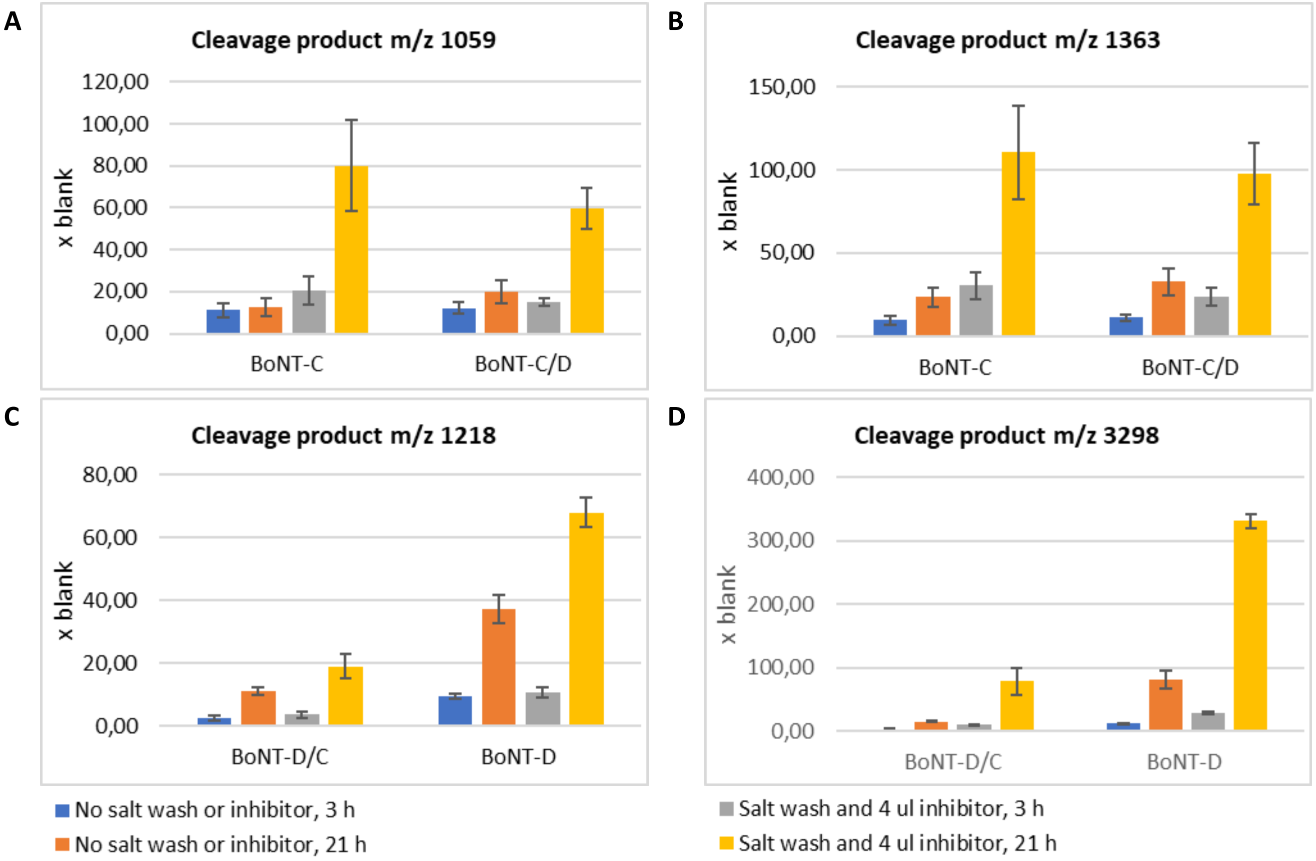

Fig. S2 Results from the method validation, using the regular and the modified Endopep-MS protocols at both 3 and 21 hours of incubation. Horse liver homogenate samples were analyzed blank and spiked with BoNT-C, C/D, D, or D/C (four replicaties each). The results for the two expected peptide cleavage products for BoNT-C and C/D are illustrated in A and B, respectively, and for BoNT-D/C and D illustated in C and D, respectively

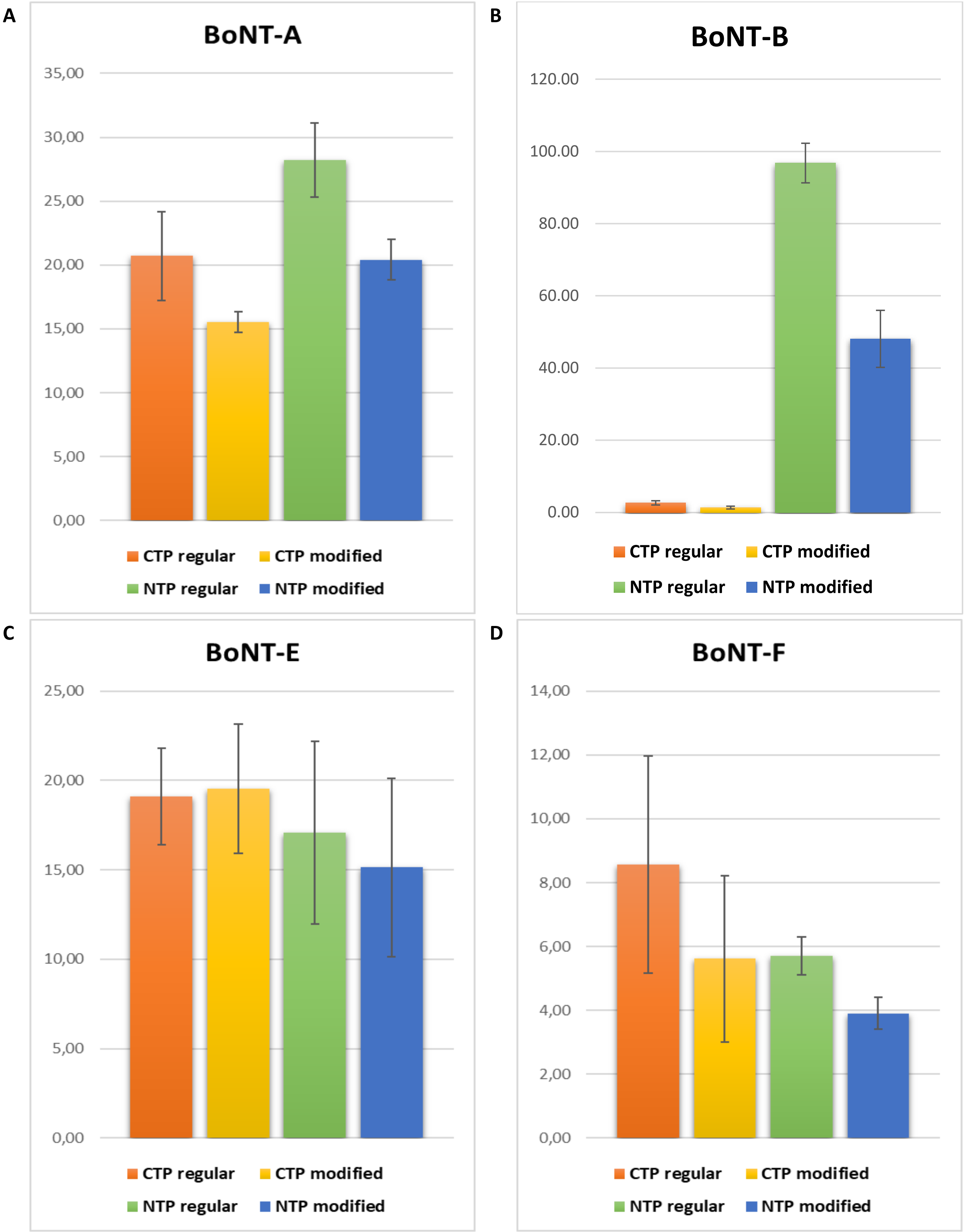

Fig. S3 Results from the method validation, using the regular and the modified Endopep-MS protocols at 3 hours of incubation. Cattle liver homogenate samples were analyzed blank and spiked with BoNT-A, B, E, and F (three replicates each). The results illustrated as x blank for the two expected peptide cleavage products, the N terminal (NTP) and the C terminal (CTP) for the peptides used for BoNT-A, B, E, and F, respectively
